# Evolution of gene expression signature in mammary gland stem cells from neonatal to old mice

**DOI:** 10.1038/s41419-022-04777-x

**Published:** 2022-04-12

**Authors:** Xiaoling Huang, Yue Xu, Lu Qian, Qian Zhao, Pengfei Liu, Jinhui Lü, Yuefan Guo, Wenjing Ma, Guangxue Wang, Shujun Li, An Luo, Xiaolai Yang, Haiyun Wang, Zuoren Yu

**Affiliations:** 1 Research Center for Translational Medicine, Shanghai East Hospital, School of Life Sciences and Technology, School of Medicine, Tongji University, Shanghai, 200120 China; 2 Department of Bioinformatics, School of Life Sciences and Technology, Tongji University, Shanghai, China; 3 Department of Pharmacy, The People’s Hospital of Gansu Province, Lanzhou, China; 4 Present Address: Department of Pharmacy, The First Affiliated Hospital of Fujian Medical University, Fujian, China

**Keywords:** Breast cancer, Ageing, Cancer stem cells, Mammary stem cells

## Abstract

During the lifetime of females, mammary epithelial cells undergo cyclical expansion and proliferation depending on the cyclical activation of mammary gland stem/progenitor cells (MaSCs) in response to the change of hormone level. The structural shrink of mammary duct tree and the functional loss of mammary gland occur along with inactivation of MaSCs in old females, even leading to breast cancer occasionally. However, the gene expression signature in MaSCs across the lifespan remains unclear. Herein, we tested the tissue regeneration ability of CD24^+^CD49f^high^ MaSCs over six time points from neonatal (4-day-old) to aged mice (360-day-old). Further RNA-seq analyses identified four clusters of gene signatures based on the gene expression patterns. A subset of stemness-related genes was identified, showing the highest level at day 4 of the neonatal age, and the lowest level at the old age. We also identified an aging-related gene signature showing significant change in the old mice, in which an association between aging process and stemness loss was indicated. The aging-related gene signature showed regulation of cancer signaling pathways, as well as aging-related diseases including Huntington disease, Parkinson disease, and Alzheimer disease. Moreover, 425, 1056, 418, and 1107 gene variants were identified at D20, D40, D90, and D180, respectively, which were mostly reported to associated with tumorigenesis and metastasis in cancer. In summary, the current study is the first to demonstrate the gene expression shift in MaSCs from neonatal to aging, which leads to stemness loss, aging, aging-related diseases, and even breast cancer in old mice.

## Introduction

In mice, mammary gland development occurs between E10.5 and E18.5 in the embryo [1]. After birth, the mammary gland epithelium remains quiescent until puberty when the mammary epithelial cells proliferate and expand into the fat pad under the stimulation of hormones and other growth factors [2]. In adult virgin mice, branching morphogenesis in the mammary gland is fully processed, where the ductal epithelium contains an outer layer of basal epithelial cells and an inner layer of luminal epithelial cells. They keep a balance between proliferation and apoptosis to maintain the epithelial branching structure, and get prepared for pregnancy and lactation. Along with growing old, the proliferation capacity of the epithelial cells is lower than apoptosis, leading to the structural shrink of the mammary duct tree and functional loss of the mammary gland [3].

During the lifetime of a female, the mammary gland epithelium undergoes many rounds of growth cycle, including changes of cell proliferation and cell differentiation in response to changes of hormonal levels. The cyclical expansion and proliferation of the epithelial cells indicates the maintenance of stem/progenitor cells in the mammary gland. It has been demonstrated that the basal myoepithelial cells in the mammary gland harbors stem/progenitor cell population [4]. Mammary stem cells (MaSCs) in mice have been isolated from lineage^-^ mammary cells by multiple research groups based on the expression of several cell-surface markers, such as moderate-to-high levels of CD24, high levels of CD29, and/or high levels CD49f [5–7]. These cells show multipotent and self-renewing, and carry ability to reconstitute complete mammary gland in vivo after transplantation [5–7]. Nonetheless, a recent publication found that the basal myoepithelial cells expressing α-smooth muscle actin were the only cell type functioning as stem cells in adult mice [8], which were characterized with the high level of epithelial cell adhesion molecule (EpCAM) and alpha 6 integrin (CD49f). So the cells with lineage^-^ CD24^+^ CD49f^high^ features were considered as MaSCs in the current study.

DNA changes happen over the period of a lifetime, which often determine the changes of relevant phenotypes. There are two types of DNA changes, one is inherited from parents, the other comes from natural aging or exposure to toxic chemicals/rays in the environment. Gene mutations in BRAC1, BRCA2, ErbB2, PTEN, TP53, et al., have been well demonstrated to cause breast cancer in women [9–12]. Along with growing old, there is consequently accumulation of such gene mutations in somatic cells, leading to human cancer occurrence [13].

Breast cancer is the most common cause of cancer death among women all over the world [14]. One in eight women in the USA develops breast cancer in her lifetime [15]. The median age of diagnosis with breast cancer in women is 62. Only ~4% of women diagnosed as breast cancer are <40 years old. Aging is the most significant risk for breast cancer. Notably, gene mutations in tissue stem cells have been considered as the main reason leading to formation of cancer stem cells, and tumorigenesis [16–18].

Since many dysregulated pathways regulating breast cancer progression are also observed during tissue development and cyclical remodeling in the mammary gland [3], identification of the gene signature upon development in the mammary gland will shed light on our understanding of the mechanisms regulating the initiation and progression of breast cancer. However, the gene expression shift in MaSCs from neonatal to adult remains unclear.

In order to determine the gene expression signature in different developmental stages of MaSCs, we herein performed genome-wide gene expression analysis based on RNA-Seq data over five time points from young to old mice, and identified multiple clusters of genes that have coherent expression patterns across the time points. Pathway analyses indicated changes of those aging-related and cancer-related genes over the lifetime in mice. Additional comparisons between the gene signatures we identified and public datasets further confirmed the evolution of gene expression in MaSCs, regulating organism growth, stemness, and aging in mammary epithelial cells (MECs), and breast tumorigenesis as well.

## Results

### Isolation of CD24^+^CD49f^high^ MaSCs from neonatal to old mice

**Fig. 1:**
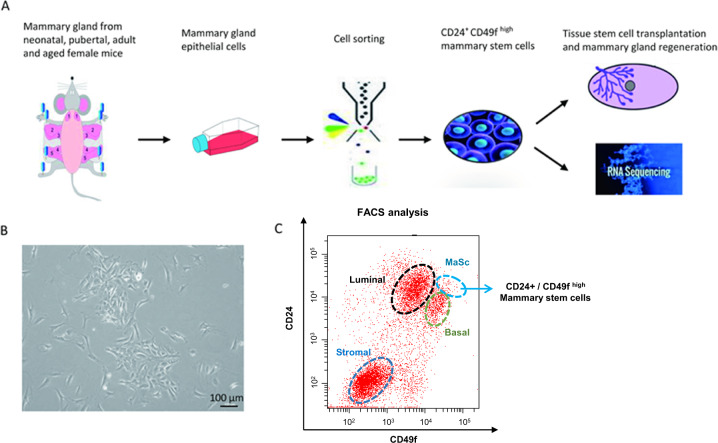
Isolation of mammary gland stem cells from mice. **A** Schematic representation of the workflow to identify the evolution of gene expression signature in the mammary gland stem cells during development from neonatal to old mice. **B** Representative primary mammary epithelial cells isolated from the mammary gland of female mice. **C** The CD24^+^CD49f ^high^ mammary stem cells were purified by cell sorting using flow cytometry.

In order to understand the evolution of gene expression in the mammary gland stem cells upon the tissue development and aging in mice, cell transplantation and RNA-seq analysis were performed with MaSCs following the procedure shown in Fig. 1A, which covered different developmental-stages of mammary gland from neonatal to aged mice. Primary MECs were isolated from the mammary glands of female mice at ages of 4, 20, 40, 90, 180, and 360 days after birth (Fig. 1B), representing developmental stages of infant, teenager, young adult, adult, early old, and old, respectively. The lineage^-^ cells at each age were sorted by flow cytometry to collect MaSCs using the CD24^+^CD49f^high^ feature and basal progenitor cells using the CD24^low^ CD49f^high^ feature (Supplemental Fig. S1 and Fig. 1C).

### Comparison of the mammary gland regenerative ability of CD24^+^CD49f^high^ MaSCs from neonatal to old mice

In order to determine the regenerative ability of the MaSCs at different developmental stages, in vitro mammosphere formation assays were performed with MaSCs from the neonatal to old mice (4-, 20-, 40-, 90-, 180- and 360-days old, respectively). MaSCs at D4 to D90 grew normally into spheres with the highest efficiency at D40, while those from D180 and D360 showed weaker sphere-formation ability, and formed smaller size of spheres (Supplemental Fig. S2A, B). Meanwhile, same assays were applied in parallel to the progenitor cells from the six age points, which did not form any spheres under the same condition. Only cell debris was observed after 8 days of culturing, suggesting the progenitor cell proliferation was not able to form mammospheres in vitro (Supplemental Fig. S2C).

**Fig. 2:**
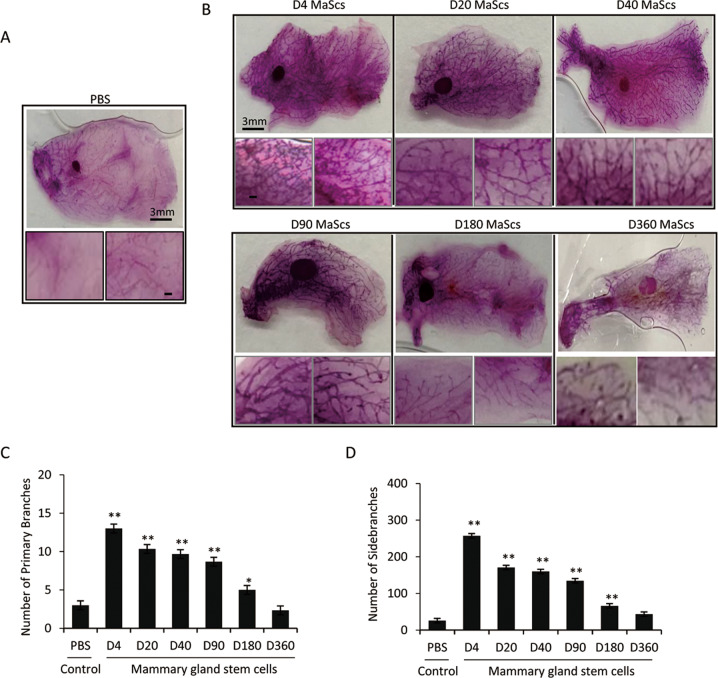
Mammary gland regeneration in vivo after mammary stem cell transplantation. **A**, **B** Whole-mount staining of the mammary branching tree structures regenerated from the mammary stem cells isolated from mice at days 4, 20, 40, 60, 180, and 360 after birth, respectively. Same volume of PBS was used for injection as a negative control (**A**). Total amount of 500 MaSCs at each time point was transplanted into the epithelium-free mammary fat pad between the inguinal lymph node and the midline of 3-week-old female mice (**B**). **C** Quantitative analysis of the primary branches extending from the lymph node to end buds. **D** Quantitative analysis of the side-branches extending from the primary branches to end buds. Data are presented as the mean ± SEM (*n* = 3). *p < 0.05, **p < 0.01.

Moreover, in vivo tissue regeneration assays were performed by transplanting MaSCs from neonatal to old mice (500 cells per injection) into the 3-week-old female mice with fat pad cleared between the nipple and the proximal lymph node. Injection of same volume of PBS served as negative controls (Fig. 2A). All the mice were checked in 10 weeks after transplantation. As shown in Fig. 2B, branching tree structures in the mammary glands were regenerated from the transplanted MaSCs. Quantitative analysis of the primary branches (ducts extending from the lymph node and terminating in an end bud) and side-branches (the branches extending from the primary branches) indicated the strong regenerative ability of MaSCs from neonatal and young mice of D4, D20, D40, and D90, while decreased at the early old age of D180, and declined to PBS control level at the old age of D360 (Fig. 2C, D).

### Gene expression profiling in the CD24^+^CD49f^high^ MaSCs from neonatal to old mice

**Fig. 3:**
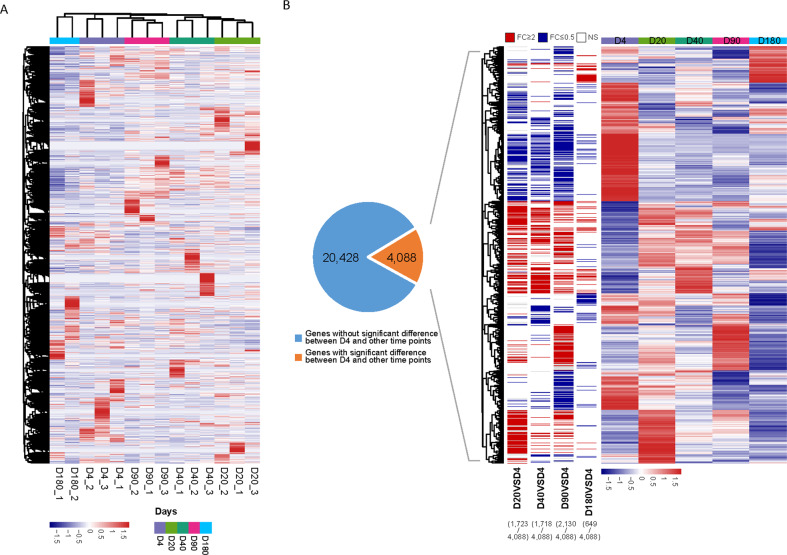
Gene expression analysis in MaSCs by RNA-seq. **A** Heatmap of 15,232 genes whose TPM values dynamically changing over the time points with standard deviation > 0.5. Hierarchical clustering algorithm was used to group samples on the basis of the expression pattern. **B** Pie chart illustrates numerical proportion of genes with or without significantly differential expression between D4 and other time points. Genes with adjusted *P*-value < 0.05 and fold change (FC) ≥ 2 were defined as statistically differential expression. Four thousand eighty-eight differentially expressed genes were represented in the heatmap.

Total RNAs from MaSCs were end linked with adapters for amplification. RNA-seq analyses were applied using Illumina sequencing platform. The abundance of each gene was quantified as TPM (Transcripts per million) value. 15,232 genes whose TPM values dynamically changing over the time points with standard deviation >0.5 were performed further analysis. Hierarchical clustering algorithm was used to group samples on the basis of the expression pattern. As expected, the repeats at each time point were clustered together (Fig. 3A). D4-based comparisons with other time points identified 4,088 differentially expressed genes (DEGs) in total (Fig. 3B).

In order to identify DEGs between adjacent time points, comparisons were made between D20 vs. D4, D40 vs. D20, D90 vs. D40, and D180 vs. D90. The DEGs were defined using adjusted *P*-value < 0.05 and fold change over 2.0 (Supplemental Fig. S3). Among them, 3128 genes were differentially expressed between D180 and D90, and 1723 between D20 and D4, but much smaller number of DEGs between D20, D40, and D90 (Supplemental Fig. S3), indicating the occurrence of the major changes in gene expression at the early stage of development (from neonatal to puberty) and aging stage (from adult to old).

**Fig. 4:**
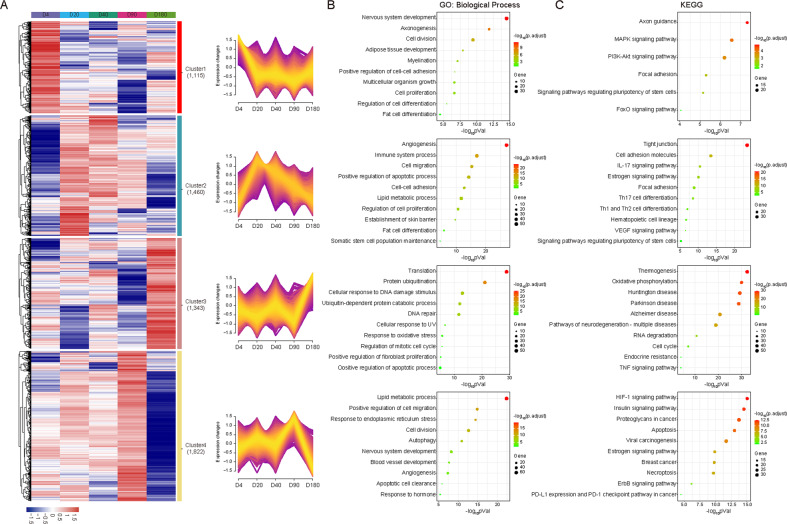
Dynamic time-series expression patterns and pathway analysis. **A** Heatmap of four clusters of differentially expressed genes identified by soft clustering representing different gene expression patterns across the five time points as indicated. **B**, **C** Gene Ontology (GO) (**B**) and Kyoto Encyclopedia of Genes and Genomes (KEGG) (**C**) enrichment analyses for the genes in the four clusters.

According to the dynamic change of gene expression over the time points, we identified four distinct patterns that summarized the expression change trends of all DEGs from neonatal to old ages by soft clustering (Fig. 4A). The genes in Cluster 1 showed the highest level at the neonatal age, and thereafter gradually downregulated. In contrast, the genes in Cluster 2 had the lowest level at D4 of the neonatal age while high level at the young adult and adult ages. Another two clusters of genes with aberrant expression at early old age of D180 were also identified, including Cluster 3 genes with the highest level at D180, and Cluster 4 genes with the lowest levels at D180.

Gene expression changes across the different time points should be the explicit representation of regulatory mechanism within cells. To assess the function of those DEGs with distinct patterns during development of the mammary gland, Gene Ontology (GO) (Fig. 4B) and KEGG (Fig. 4C) enrichment analyses were performed. As shown in Supplemental Fig. S4A, 11,144 genes had stable expression levels over the five time points, which are generally required in maintaining the tissue homeostasis and keeping balance between cell proliferation and apoptosis (Supplemental Fig. S4B, C).

Interestingly, genes in Cluster 1 with a highest level at D4 were mainly enriched in regulating cell differentiation, tissue development, signal transduction, organism growth, adipose tissue development, axongenesis, and signaling pathways regulating pluripotency of stem cells. Cluster 2 includes genes with high levels from D20 to D90, which were mainly enriched in processes of angiogenesis, somatic stem cell population maintenance, estrogen signaling pathway, and immune signaling pathway. Genes in Cluster 3 showed the highest level at the early age of D180, mainly enriched in signaling pathways regulating DNA repair, cellular apoptosis and aging-related diseases, such as Huntington disease, Parkinson disease and Alzheimer disease. In contrast to Cluster 3, genes in Cluster 4 showed the lowest level at D180, mainly enriched in hormone response signaling including estrogen, ErbB and PD-1, and pathways regulating cancer (Fig. 4C).

### Identification of the gene expression signature regulating cell stemness and/or aging

**Fig. 5:**
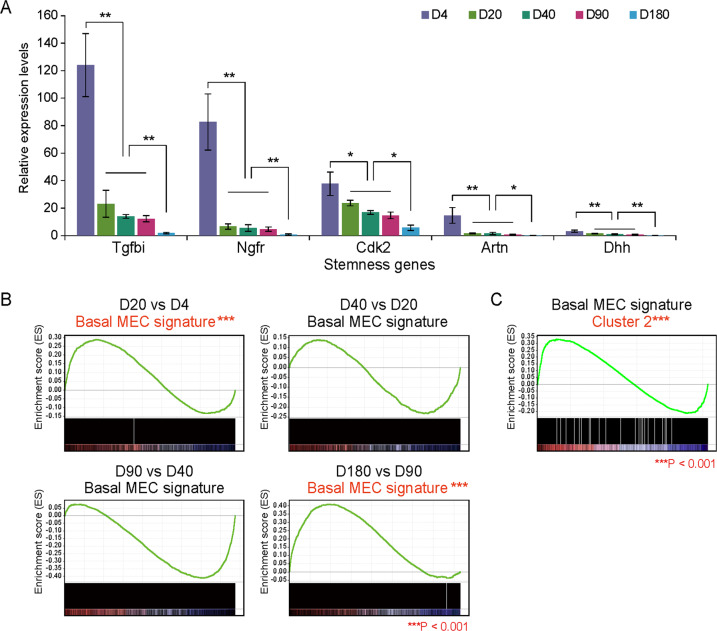
Stem cell-related gene expression signature. **A** Expression pattern of the representative stemness-related genes including TGFBI, NGFR, CDK2, ARTN, and DHH indicating the highest levels at D4 of the neonatal age and the lowest levels at the D180 of old age. **B** GSEA analysis revealed that DEGs in D20 vs. D4 and D180 vs. D90 are significantly enriched in a stemness gene signature derived from GSE116385. **C** GSEA analysis showed upregulation of DEGs in Cluster 2 in basal MECs vs. fMaSCs.

Cluster 1 includes a subset of stemness-related genes showing the highest levels at the neonatal age, and the lowest levels at the old age (Fig. 5A). In order to further confirm the gene expression signatures in MaSCs regulating stemness and/or aging, we performed comparisons between the DEGs from our dataset with the gene expression signatures in the public database. Stemness- and aging-related gene signatures were derived from Gene Expression Omnibus (GEO), including GSE116385 (an RNA-Seq dataset on normal mammary stem cell and normal adult basal cell populations [19]) and GSE130472 (an RNA-Seq dataset on aged and young mammary tumors [20]). GSEA analysis revealed that DEGs in D20 vs. D4 and D180 vs. D90 were significantly enriched with stemness genes, suggesting that the stemness change was mainly occurred at the early stage (from neonatal to puberty) and late stage (from adult to old) during mammary development (Fig. 5B). In addition, a comparison between GSE116385 (Basal MECs vs. fMaSC) and Cluster 2 (high level at the young adult and adult ages) suggested the function of this cluster of genes in regulating MEC differentiation at the age of adult in mice (Fig. 5C).

**Fig. 6:**
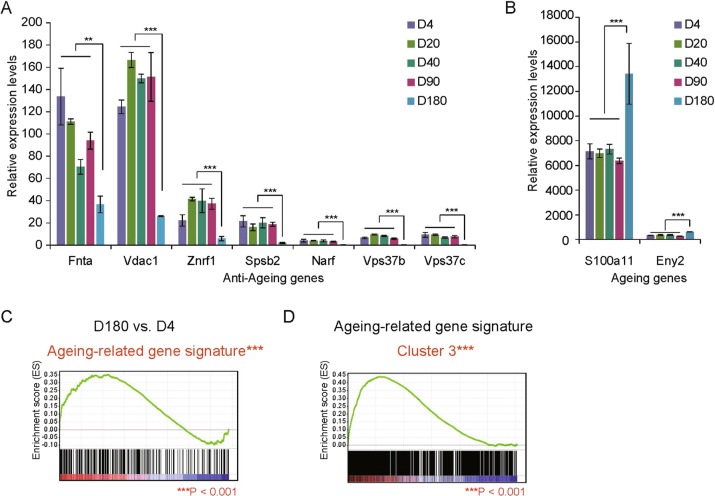
Aging-related gene expression signature. **A** Decreased expression of the representative anti-aging genes including FNTA, VDAC1, NRF1, SPSB2, NARF, VPS37B, and VPS37C at D180, compared to D4. **B** Increased expression of the aging-promoting genes S100A11 and ENY2 at D180, compared to D4. **C** GSEA analysis revealed that DEGs in D180 vs. D4 are significantly enriched in an aging-related gene signature derived from GSE130472. **D** GSEA analysis showed upregulation of Cluster 3 genes in aged vs. young mice. DEG differentially expressed gene. Data are presented as the mean ± SEM (*n* = 3). *p < 0.05, **p < 0.01, ***p < 0.001.

Cluster 3/4 include a subset of aging-related genes with the most significant change at the old age, representing the loss of stemness when getting old. The decreased expression of anti-aging genes including FNTA, VDAC1, NRF1, SPSB2, NARF, VPS37B, and VPS37C, and the increased expression of aging-promoting genes S100A11 and ENY2 at D180 were shown in Fig. 6A, B. A significant overlap between DEGs in old vs. neonatal ages and aging-related gene signature in GSE130472 further demonstrated an association between aging process and stemness loss in the old mice (Fig. 6C). GSEA analysis demonstrated consistency between genes in Cluster 3 and GSE130472 showing the high expression level at the old age (Fig. 6D).

### Identification of the gene expression signature relating tumorigenesis in old mice

**Fig. 7:**
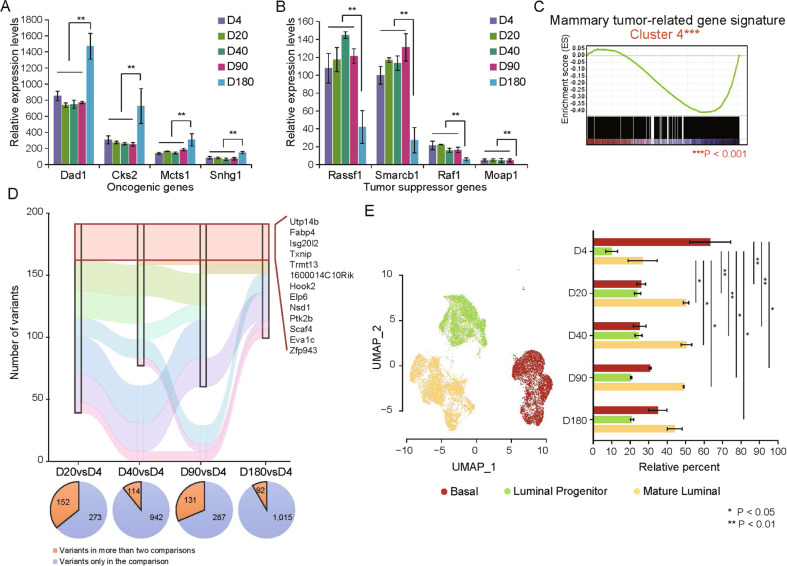
Mammary gland tumor-related gene expression signature. **A**, **B** Increased expression of the representative oncogenes including DAD1, CKS2, MCTS1, and SNHG1 (**A**), and decrease of tumor suppressor genes including RASSF1, SMARCB1, RAF1, and MOAP1 (**B**) at D180, compared to any younger time points. **C** GSEA analysis revealed that genes in Cluster 4 are significantly enriched in a mammary gland tumor-related gene signature derived from GSE130472. **D** Gene mutations at different time points compared to D4 were called by GATK and Varscan2. Sankey diagram showed the number of variants at different time points, in which different colors represent different mutation patterns. The frequency of mutations at each time point was indicated below the Sankey diagram. **E** Analysis using a cell type-based signature derived from GSE130472 (left panel) indicated the highest proportion of basal cells at D4. While both luminal progenitor and mature luminal cells showed the lowest proportion at D4, and maintained at a high level from D20 to D180 (right panel). DEG differentially expressed gene. Data are presented as the mean ± SEM (*n* = 3). *p < 0.05, **p < 0.01, ***p < 0.001.

In order to understand the relationship between aging and tumorigenesis in mammary gland, the DEGs in the Cluster 3 and Cluster 4 were further analyzed. Figure 7 showed the increased expression of oncogenes at the early old age, such as DAD1, CKS2, MCTS1 and SNHG1 (Fig. 7A), and decreased expression of tumor suppressor genes, such as RASSF1, SMARCB1, RAF1 and MOAP1 (Fig. 7B). In addition, a significant overlap between the gene expression signature in GSE130472 (your and old mammary gland tumors) and DEGs in Cluster 4 further supported the involvement of aging-related genes in mammary gland tumorigenesis (Fig. 7C).

In view of the gene mutation accumulation which usually causes tumorigenesis, we called the gene variants using the RNA-seq data via GATK and Varscan2 [21]. Setting gene sequence at D4 as a reference, there were 425, 1056, 418, and 1107 variants identified at D20, D40, D90, and D180, respectively, in which 152, 114, 131, and 92 variants were observed in more than two comparisons. Sankey diagram indicated a subset of somatic variants with persistent identification at D20, D40, D90, and D180. The majority of these mutant genes have been reported to associate with tumorigenesis and metastasis of cancer cells (Fig. 7D), such as TXNIP in TNBCs and NSD1 in renal cell carcinoma.

In addition, using a cell type signature derived from the single-cell RNA-Seq dataset GSE130472 [22], we tracked changes of the cell type-based signature from neonatal to old via a machine learning tool CIBERSORTx. As a result, basal cell signature showed the highest level at D4, while both luminal progenitor signature and mature luminal cell signature showed the lowest level at D4 and maintained at a high level from D20 to D180 (Fig. 7E), which is consistent with the stemness characteristics of mammary basal cells.

## Discussion

In consideration of the growth cycle including the cyclical expansion and proliferation of the epithelial cells, the mammary gland provides a unique mode for studying developmental process, tissue regenerative property, and tissue stem cell evolution. It is the MaSCs that drive the development of mammary gland and maintain the regenerative capability of epithelium. So identification of the gene expression signatures in MaSCs will lead to our better understanding of the mechanisms in regulation of the cyclical architecture, as well as the epithelium shrink and tumorigenesis in the mammary gland of old mice.

Considering the key role of estrogens in influencing the morphological changes of the mammary gland throughout the estrous cycle and fertility in homeostasis, as well as the role of estrogens in tumorigenesis, cytology in vaginal smears has been applied to determine the stages of the estrous cycle in the female mice at different developmental stages. All the female mice in the current study were separated from males after birth, the estrous cycle in these mice were determined by the level of hormones, such as estrogen. Since the mice we used at the different ages were selected in random, vaginal cytology examination did not show a definite stage for the specific ages of the mice we used.

In the current study, through evaluation of the gland regeneration after cell transplantation in vivo, we confirmed that the regenerative potential of CD24^+^CD49f^high^ MaSCs is strong in the young mice, while decline in the mice when getting old. This observation is consistent with the developmental-stage associated function of mammary gland. Moreover, functional analyses on the gene expression signatures in MaSCs we identified from neonatal to old mice are in supportive of the status, structure, and function of the mammary epitheliums corresponding to the developmental stages.

Genes in Cluster 1 are mainly involved in cell differentiation, tissue development, and signal pathways regulating organism growth, adipose tissue development, and fat cell differentiation. They had the highest expression level at D4, and decreased at D20 and thereafter, such as NGFR and ARTN (Fig. 5A), which have been reported to regulate stem cells and/or progenitor cells [23–25]. Moreover, we found the highest cell proportion of MaScs at D20 and D40 (Supplemental Fig. S1), although MaScs at D4 had the strongest tissue regenerative ability (Fig. 2). Since gene p53 and its homolog p63 have been implicated to regulate mammary stem cell self-renewal, we analyzed the expression of p53 and p63 in MaScs across the time points from neonatal to old mice. In consistence the result in Fig. S1, p53 and p63 showed higher levels at D20 and D40 (Supplemental Fig. S5A, B), supporting the developmental regulation of mammary epithelium and increased self-renewing of MaScs during puberty and young adult stages in mice.

The decrease of anti-aging genes and increase of aging-promoting genes at the old age (Fig. 6A, B) confirmed the hypothesis that the gene expression changes determine the cell/tissue aging, even aging-related diseases. For example, anti-aging genes NRF1 and SPSB2 showed expressional loss, while aging-promoting genes S100A11 and ENY2 showed significant increase at the early old age. These genes have been demonstrated to involve in the regulation of aging or aging-related diseases [26–29].

Accumulation of genetic mutations and epigenetic alterations in tissue stem/progenitor cells, or even in the differentiated cells are supposed to be the source to induce the formation of cancer stem cells (also known as tumor-initiating cells) [30]. The current study well supported the hypothesis that the alterations of tissue stem/progenitor cells may be the origin of cancer stem cell formation. In addition to gene mutation, aging is considered as the most significant risk for cancer, especially for breast cancer and prostate cancer. Herein, we found the gene expression signature in the old mice is enriched in the signaling pathways regulating aging-related diseases including Huntington disease, Parkinson disease, and Alzheimer disease. Based on these findings, the gene signature we identified in the old mice takes responsibility to a certain extent for those aging-related diseases including cancer.

## Materials and Methods

### Animals

Animal studies were approved by the Institutional Animal Care and Use Committee of the Tongji University School of Medicine. FVB mice were purchased from the Silaike Animal Company (Shanghai, China). Isolation of the mammary gland epithelial cells from mice was performed in the Research Center for Translational Center, Tongji University School of Medicine. All experiments were performed in accordance with the relevant guidelines and regulations for animal use.

### Isolation of mammary gland epithelial cells

Mammary glands were dissected from different ages of female mice to isolate epithelial cells following the protocol from literature [4, 5] with a small modification. Briefly, after mechanical dissociation, the tissue pieces were placed in culture medium (DMEM with 1 mM glutamine, 5 mg/ml insulin, 500 ng/ml hydrocortisone, 10 ng/ml epidermal growth factor, and 20 ng/ml cholera toxin, 5% bovine calf serum) containing 300 U/ml collagenase and 100 U/ml hyaluronidase for 1 h at 37 °C. The resultant organoid suspension was sequentially resuspended in 0.25% trypsin-EDTA containing 5 mg/ml Dispase for 5 min before filtration through a 40-mm mesh. The cells were cultured for 12–24 h to attach to the culturing dishes. The fibroblasts were removed through differential adhesion method. The enriched mammary epithelial cells at this step were in passage 0 to passage 1, still maintaining the primary cell property and function. Immediately after enrichment, MECs were applied for Lineage negative (Lin-) selection following a widely used strategy for purifying mammary epithelial cells (CD31^−^CD45^−^Ter119^−^).

### Isolation of mammary gland stem cells

Lin- mammary gland epithelial cells were suspended in the medium containing antibodies against CD24 (conjugated to PE, 1: 100, Cat# 60099PE.1, Stemcell Technologies, Canada) and CD49f (conjugated to APC, 1: 20, #130100147, Miltenyi Biotec, USA), and incubated in the dark cold room at 4 °C for 20 min. After washing twice with 1× PBS, the cells were centrifugated at 200 × g for 5 min. Discarding the supernatant, and resuspending the cell pellet in 300 µl 1× PBS. The labeled mammary gland stem cells were sorted using a FACStarPLUS (BD Biosciences, USA) flow cytometer. Data were analyzed with CytExpert software. Following the approach, mammary gland stem cells were obtained from female mice at the ages of day 4 (D4), day 20 (D20), day 40 (D40), day 90 (D90), day 180 (D180), and day 360 (D360), respectively.

### Mammosphere formation assay

Mammary gland stem cells were planted into 96-well ultra-low adherent cell culture plate (Corning, USA) with the density of 500 cells/well, and incubated at 37 °C and 5% CO2 for 1 week in DMEM/F12 medium containing 20 ng/mL bFGF, 20 ng/mL EGF, 10 μg/mL heparin, 10 μg/mL insulin, 1% Penicillin-Streptomycin solution, and B27 supplement (Invitrogen). Sphere photos were captured at day 1, day 4, and day 7 with Axio Vert A1 FL microscope (Zeiss).

### Transplantation of mammary stem cells into the fat pad of mice

Three-week-old female mice were used for cell transplantation following the protocol described in literature [31]. Briefly, after anesthetization, cauterizing the mammary artery running between the fourth/fifth mammary fat pads of mice and the two blood vessels around the proximal lymph node. Then cutting the bridge between fourth and fifth mammary glands to prevent the epithelium growing in the fourth mammary fat pads from the fifth, and removing the region of the fourth mammary fat pad between the nipple and the proximal lymph node. Five hundred of the sorted Lin^−^ CD24^+^ CD49f^high^ mammary gland stem cells were injected into the remaining portion of the epithelium-free mammary fat pad between the inguinal lymph node and the midline. For the cell recipients, three randomly selected female mice were applied for each time point of MaScs without blinding to investigators. An online tool (www.random-online.com) was used for randomization. In 10 weeks post transplantation, mammary outgrowths were collected and applied for whole-mount staining.

### Whole-mount staining of mouse mammary gland

The fourth mammary gland was taken from a mouse, and spread out on a glazed microscope slide. Then immediately fixed in the fresh 4% formaldehyde for 4 h. After washing in PBS twice for 5 min each, the Carmine Alum solution was applied for staining overnight on a rocking bed at room temperature. After washing with 50, 75, 85, 95, and 100% of EtOH in order for 30 min each, clear the mammary gland in xylene and mount with permount under a coverslip. Stained mammary glands were imaged by light microscopy.

### RNA sequencing (RNA-seq) analysis

A pool of 500 sorted mouse mammary gland stem cells at an indicated stage were treated with the lysis buffer to isolate total RNA, and applied to reverse transcription synthesis of the first-strand cDNA and further amplification of double-strand cDNA using the Discover-scTM WTA Kit V2 (Vazyme, China) following the manufacturer’s instructions (a detailed flow diagram in Supplemental Fig. S1). VAHTSTM DNA Clean Beads (Vazyme) were used for size-selection and purification of the amplified cDNA library. After quality validation using Agilent 2100 Bioanalyzer (Applied Biosystems, USA), the library was sequenced (*n* = 3) by the HiSeq System (Illumina, USA).

### RNA-seq data processing

The quality of the reads was evaluated with FastQC (version 0.11.6) [32]. We applied trim_galore (version 0.4.4) [33] and cutadapt (version 1.16) [34] to remove the adapters and overrepresented sequence. The samples information of clean data was shown in Supplemental Table S1.

The paired-end clean RNA-seq reads were aligned to the mouse reference Ensembl Version GRCm38.92 using the splice-aware aligner STAR (v2.4.0j) [35]. The abundance of each gene was quantified as TPM (Transcripts per million) value, which was evaluated by a statistical method RSEM (RNA-Seq by Expectation Maximization) using a generative model of RNA-seq reads and the EM algorithm, taking read mapping uncertainty into account and achieving the most accurate abundance estimates [36].

### Hierarchical clustering, heatmap visualization

We calculated the standard deviation (SD) of each gene over five time points, and used cut-off with SD ≥ 0.5 genes to generate a hierarchical clustering and heatmap with the “pheatmap” package in R.

### Dynamic expression analysis

The analysis of differentially expressed genes (DEGs) was performed using the DESeq2 [37] with raw counts of RNA-seq data. Adjusted *p*-values corrected with the Benjamini–Hochberg method <0.05 and fold change over 2.0 were set as the cutoff for controlling false positives. The dynamic expression patterns were identified by soft clustering using Mfuzz with the parameters: numbers of clusters (*c*) = 4, fuzzification (*m*) = 2.0 [38]. Genes in four clusters were further mapped onto the Gene Ontology (GO) as well as Kyoto Encyclopedia of Genes and Genomes (KEGG). Adjusted *P*-values were calculated using the hypergeometric distribution.

### The public datasets in GEO Database and DEGs analysis

Three gene expression datasets in Gene Expression Omnibus (GEO, http://www.ncbi.nlm.nih.gov/geo/) database were used, including GSE116385 (a RNA-Seq dataset on normal mammary stem cell populations and fetal mammary stem cell populations [19]), GSE130472 (an RNA-Seq dataset on aged and young mammary tumors [20]) and GSE103275 (a single-cell RNA-Seq dataset on mouse mammary epithelial cells [22]).

For the bulk RNA-Seq data GSE116385 and GSE130472, the raw counts were downloaded from GEO database, and DEGs with adjusted *P*-value < 0.05 and fold change ≥ 2 were detected by DESeq2. For GSE116385, 1567 genes were significantly upregulated and 1303 genes downregulated in the comparison of fMaSC vs. Basal MECs. For GSE130472, 405 genes were significantly upregulated and 910 downregulated in the comparison of old vs. young mammary tumors.

### Gene Set Enrichment Analysis (GSEA)

GSEA [39] was used to further analyze if the gene expression changes in the mammary stem cells over time points were associated with the gene signatures of stemness, aging, and/or cancer, which were derived from the public datasets as mentioned above. The DEGs of four comparisons, D20 vs. D4, D40 vs. D20, D90 vs. D40, D180 vs. D90, were respectively compared with the mammary development, aging, and/or cancer gene signatures, and the family-wise error rate was used for significance correction to reduce false positive. In addition, the signatures derived from the dynamic expression patterns were validated in the public databases using GSEA.

### Mutation calling for RNA-Seq

STAR two-pass method was used to perform alignments to the mouse reference Ensembl Version GRCm38.92. Duplicates were marked using Picard’s Mark Duplicates tool (Version 2.0.1). Reads were split and trimmed using the Genome Analysis Toolkit (GATK, version 4.0) SplitNTrim tool, after which indel realignment and base recalibration were performed. The sorted alignment files were then merged by samtools [40]. A pileup file was created using the final recalibrated bam file and samtools mpileup. Finally, variants were called using Varscan2 (v2.4.4) [21] in the comparisons of D20 vs. D4, D40 vs. D4, D90 vs. D4, and D180 vs. D4. Somatic variants were further selected to increase the confidence by Varscan2 processSomatic. Functional annotation was performed using SNPeff [41]. Known RNA editing sites collected by REDIportal were excluded for further analysis [42]. Finally, we created a Sankey diagram to visualize the variant distribution between different time points.

### Estimation of cell fractions

For the assessment of cellular abundance and cell type-specific gene expression patterns from bulk tissue transcriptome profiles, CIBERSORTx was used to evaluate the relative percentages of stem cells at different time points, and determine the changes of cell abundance by leveraging cell type expression signatures derived from single-cell RNA-Seq data [43].

### Statistical analysis

Data are presented as mean ± SEM unless stated otherwise. The standard two-tailed student’s *t*-test was used for statistical analysis, in which *p* < 0.05 was considered significant.

## Supplementary information

Supplemental Data

Reproducibility checklist

## Data Availability

Original data presented in this study are included in the article/supplementary information. Further inquiries are available upon request to the corresponding authors.
