# Supplementary material for: Evolution of gene expression signature in mammary gland stem cells from neonatal to old mice
Source: Cell Death Dis. 2022 Apr 12;13(4):335. doi: 10.1038/s41419-022-04777-x (PMC9001724; doi:10.1038/s41419-022-04777-x)

Supplemental Information

Supplemental Table S1: The clean reads information of all samples by RNA-seq analysis.

Supplemental Figure S1. A: Isolation of MaScs at the six time points (D4, D20, D40, D90, D180 and D360) by cell sorting with flow cytometry. The $CD24^{+}$ $CD49f^{high}$ MaScs and $CD24^{low}$ $CD49f^{high}$ basal (progenitor) cell populations were labeled with squares. **B:** Quantitative analysis of the $CD24^{+}$ $CD49f^{high}$ MaScs . **C:** Quantitative analysis of the $CD24^{low}$ $CD49f^{high}$ progenitor cells. The experiments were repeated three times independently. Data are presented as the mean ± SEM (n=3). **p<0.01. All comparisons were *vs* D4.

Supplemental Figure S2. Mammosphere formation assays with MaSCs (**A, B**) and progenitor cells (**C**) from the mice of the six time points (D4, D20, D40, D90, D180 and D360). The sphere photos were captured at day 0 and day 8 after culturing. The size (diameter) of the spheres were quantified, and presented as the mean ± SEM (n=10).**p<0.01. All comparisons were *vs* D4.

Supplemental Figure S3. Differentially expressed genes (DEGs) between the successive time points including D20 vs. D4, D40 vs. D20, D90 vs. D40, and D180 vs. D90. Genes with the adjusted P-value < 0.05 and FC >= 2 were defined as statistically differentially expressed ones.

Supplemental Figure S4. Pattern plot of the 11,144 genes with stable expression over the five time points (A). GO Biological Process (B) and KEGG (C) analyses were performed based on hypergeometric distribution (adjusted P-value < 0.05).

Supplemental Figure S5. Expression of p53 and p63 in MaSCs at the six time points (D4, D20, D40, D90, D180 and D360) by QRT-PCR analysis. Data are presented as the mean ± SEM (n=3). *p<0.05, **p<0.01. All comparisons were *vs* D4.

Table S1 The information of the samples

Sample name	Sample fastq	# of clean reads
D4_1	VZ20180119-03-R70870-01_HHMLJCCXY_L4_1.fastq	26,905,418
	VZ20180119-03-R70870-01_HHMLJCCXY_L4_2.fastq	26,905,418
D4_2	VZ20180119-03-R70870-02_HHMLJCCXY_L4_1.fastq	27,764,448
	VZ20180119-03-R70870-02_HHMLJCCXY_L4_2.fastq	27,764,448
D4_3	VZ20180119-03-R70870-03_HHMLJCCXY_L4_1.fastq	24,711,523
	VZ20180119-03-R70870-03_HHMLJCCXY_L4_2.fastq	24,711,523
D20_1	VZ20180119-03-R70870-3-01_HHMLJCCXY_L4_1.fastq	23,182,198
	VZ20180119-03-R70870-3-01_HHMLJCCXY_L4_2.fastq	23,182,198
D20_2	VZ20180119-03-R70870-3-02_HHMLJCCXY_L4_1.fastq	26,237,202
	VZ20180119-03-R70870-3-02_HHMLJCCXY_L4_2.fastq	26,237,202
D20_3	VZ20180119-03-R70870-3-03_HHMLJCCXY_L4_1.fastq	23,530,449
	VZ20180119-03-R70870-3-03_HHMLJCCXY_L4_2.fastq	23,530,449
D40_1	VZ20180119-03-R70870-07_HHMLJCCXY_L4_1.fastq	27,961,665
	VZ20180119-03-R70870-07_HHMLJCCXY_L4_2.fastq	27,961,665
D40_2	VZ20180119-03-R70870-08_HHMLJCCXY_L4_1.fastq	25,813,256
	VZ20180119-03-R70870-08_HHMLJCCXY_L4_2.fastq	25,813,256
D40_3	VZ20180119-03-R70870-2-06_HHMLJCCXY_L4_1.fastq	27,534,144
	VZ20180119-03-R70870-2-06_HHMLJCCXY_L4_2.fastq	27,534,144
D90_1	VZ20180119-03-R70870-2-07_HHMLJCCXY_L4_1.fastq	24,786,554
	VZ20180119-03-R70870-2-07_HHMLJCCXY_L4_2.fastq	24,786,554
D90_2	VZ20180119-03-R70870-2-08_HHMLJCCXY_L4_1.fastq	27,016,801
	VZ20180119-03-R70870-2-08_HHMLJCCXY_L4_2.fastq	27,016,801
D90_3	VZ20180119-03-R70870-2-09_HHMLJCCXY_L4_1.fastq	27,994,521
	VZ20180119-03-R70870-2-09_HHMLJCCXY_L4_2.fastq	27,994,521
D180_1	VZ20180119-03-R70870-10_HHMLJCCXY_L4_1.fastq	22,413,754
	VZ20180119-03-R70870-10_HHMLJCCXY_L4_2.fastq	22,413,754
D180_2	VZ20180119-03-R70870-12_HHMLJCCXY_L4_1.fastq	24,704,161
	VZ20180119-03-R70870-12_HHMLJCCXY_L4_2.fastq	24,704,161
D180_3	VZ20180119-03-R70870-3-05_HHMLJCCXY_L4_1.fastq	24,924,582
	VZ20180119-03-R70870-3-05_HHMLJCCXY_L4_2.fastq	24,924,582

Supplemental Figure S1

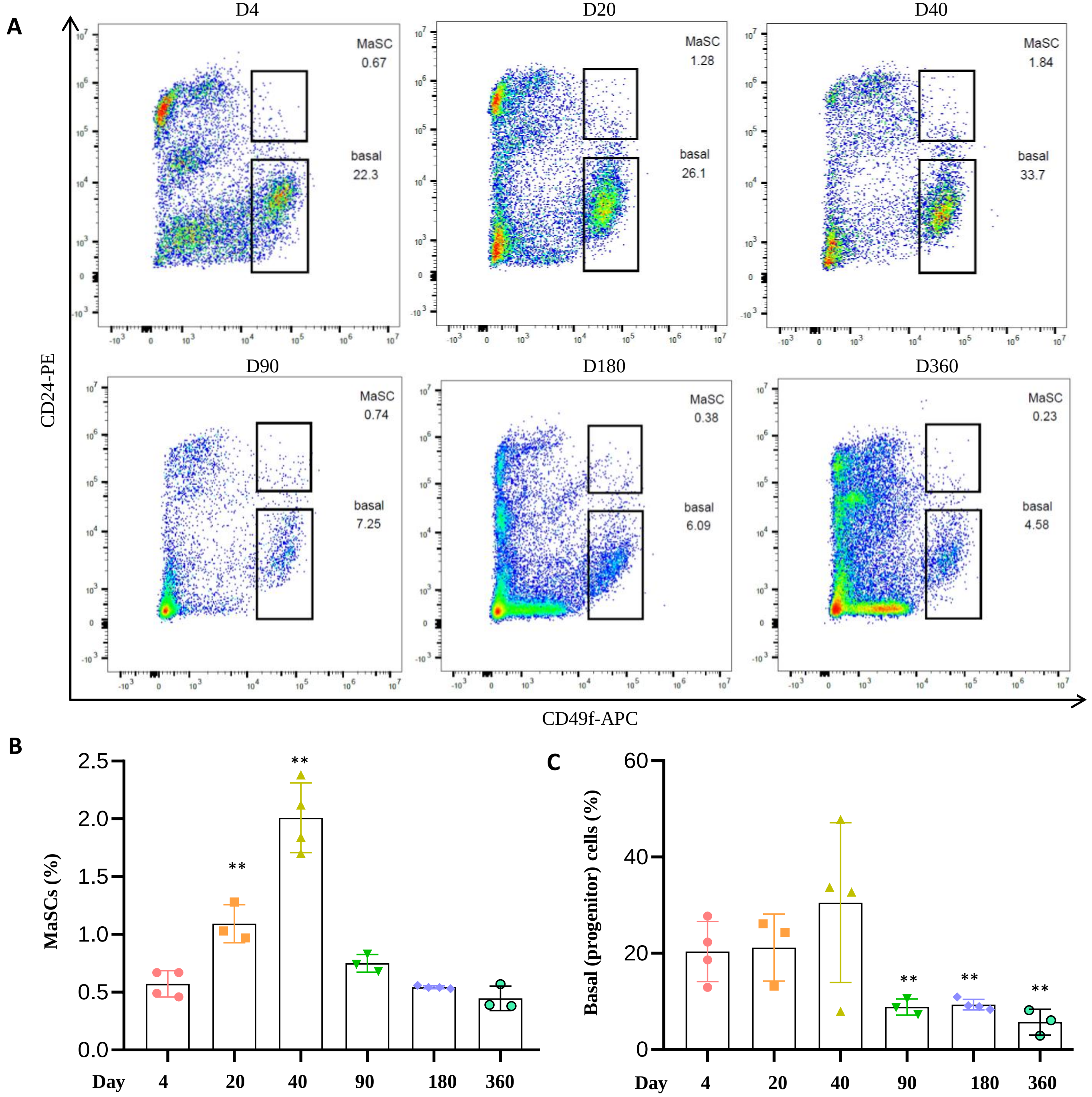

Supplemental Figure S2

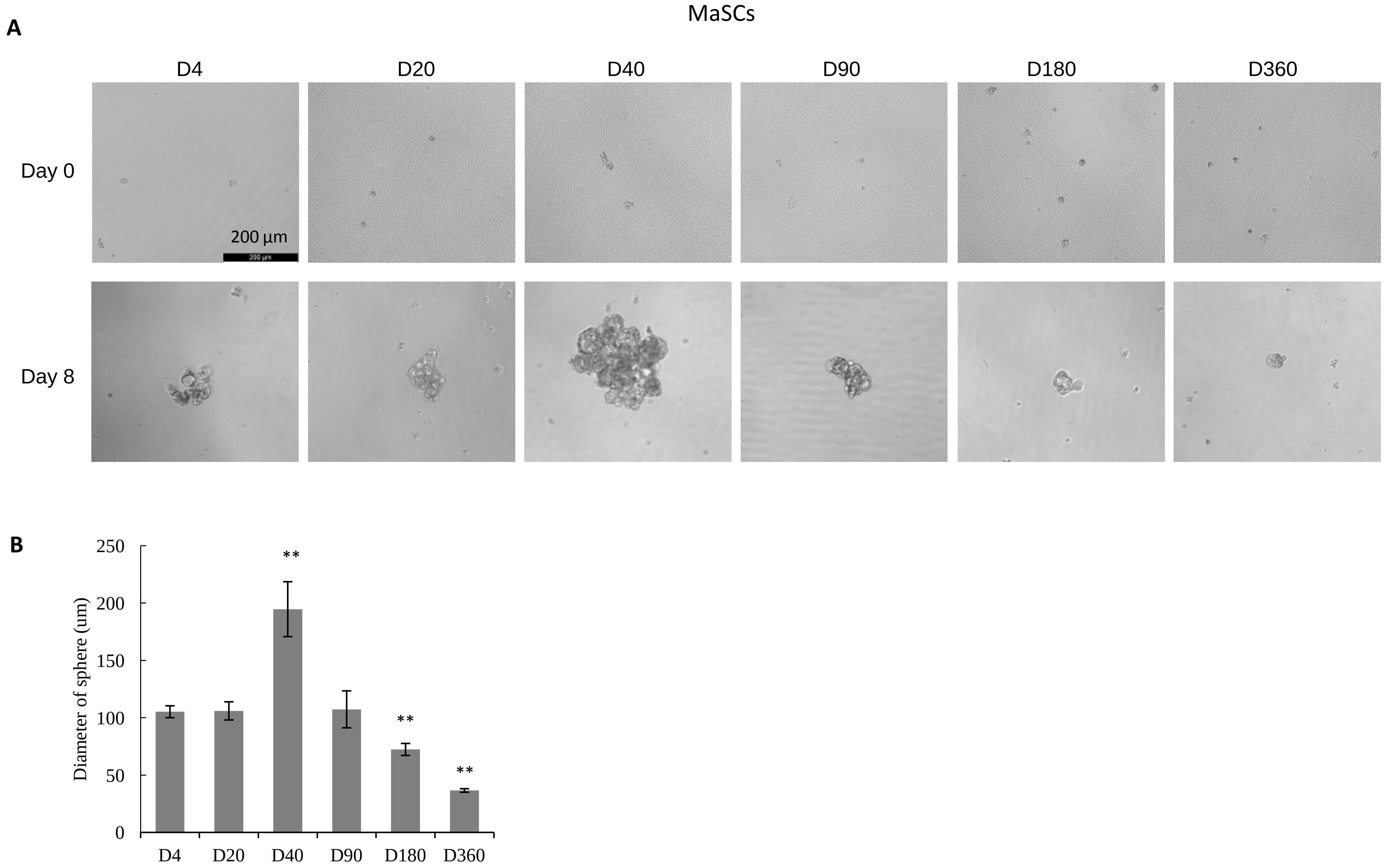

Supplemental Figure S2

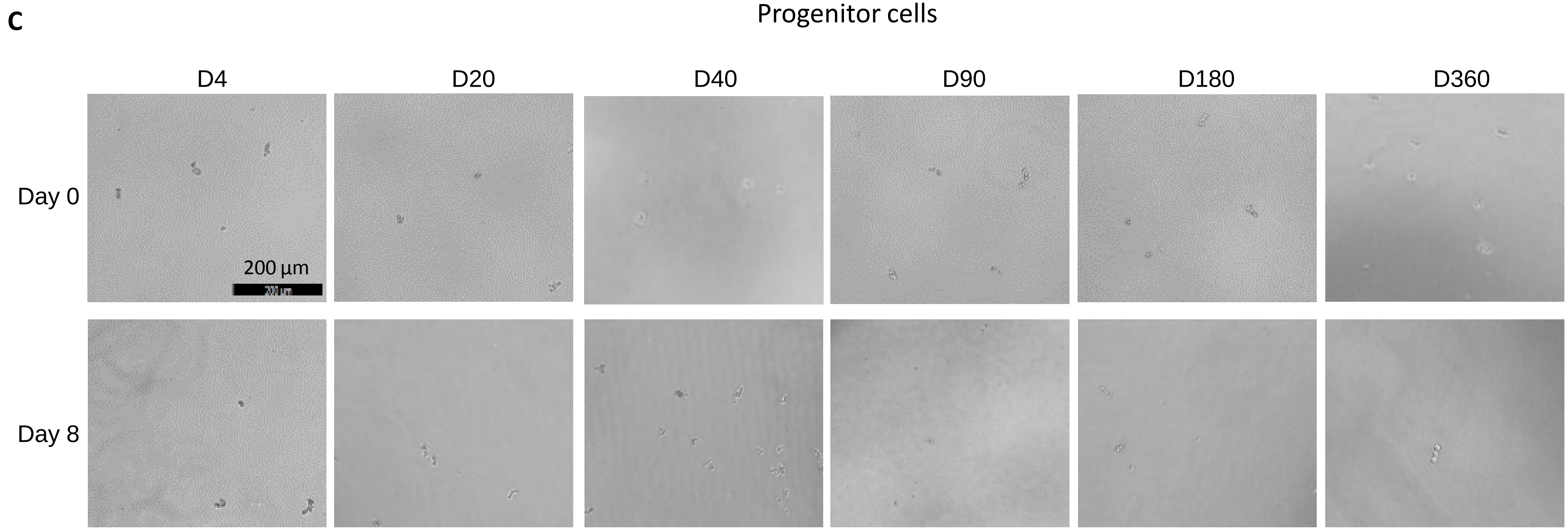

Supplemental Figure S3

D20 VS D4

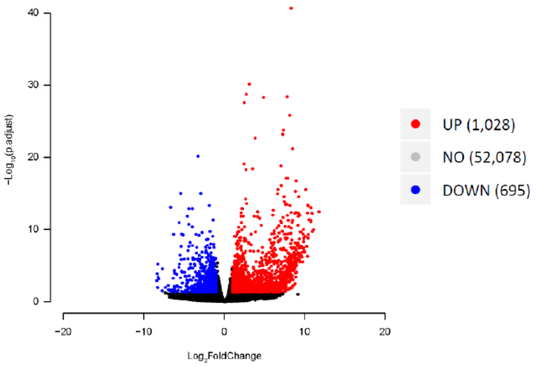

D40 VS D20

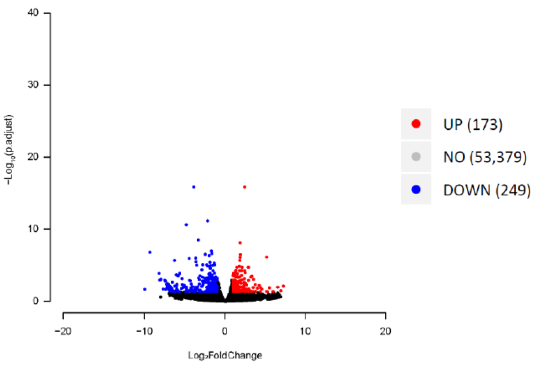

D90 VS D40

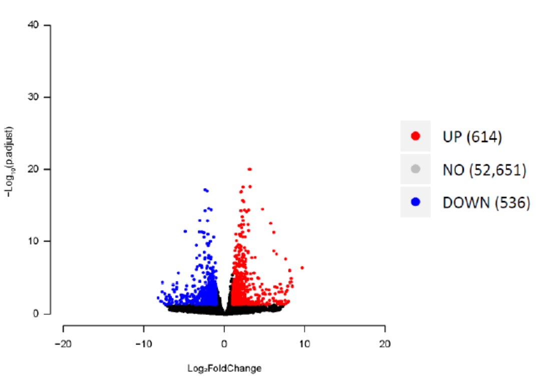

D180 VS D90

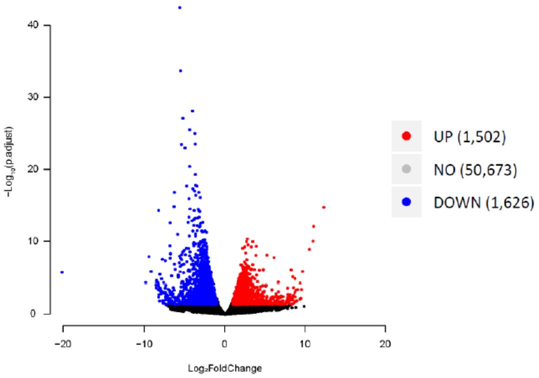

Supplemental Figure S4

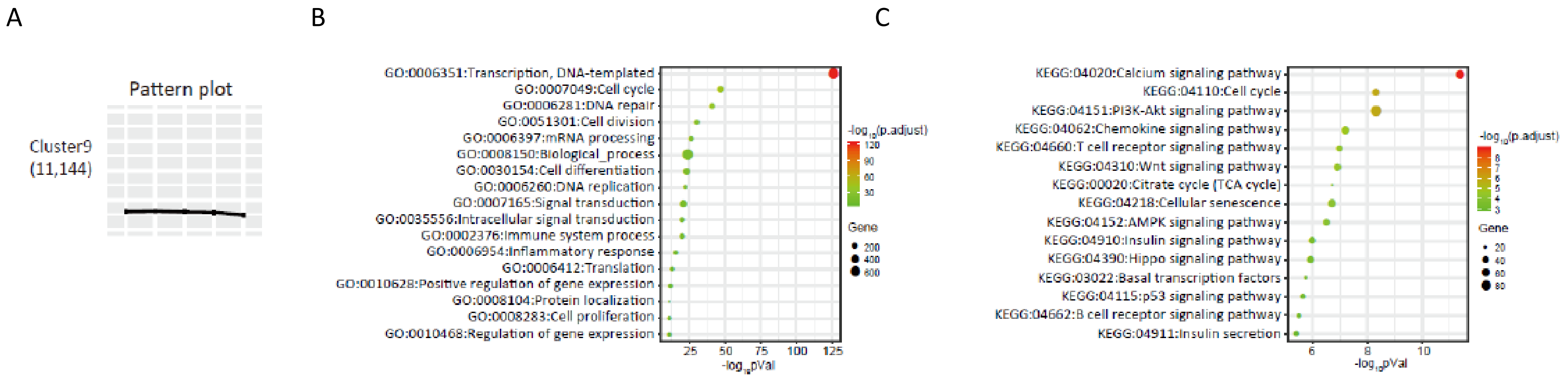

Supplemental Figure S5

A

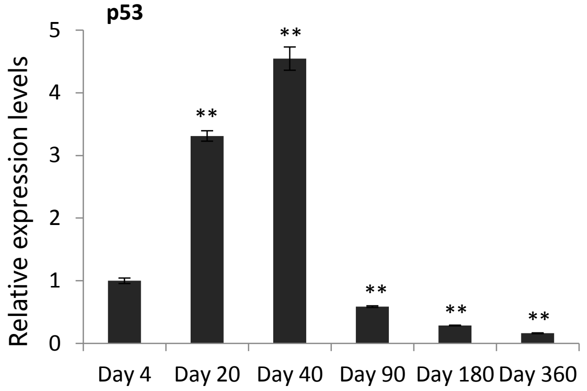

B

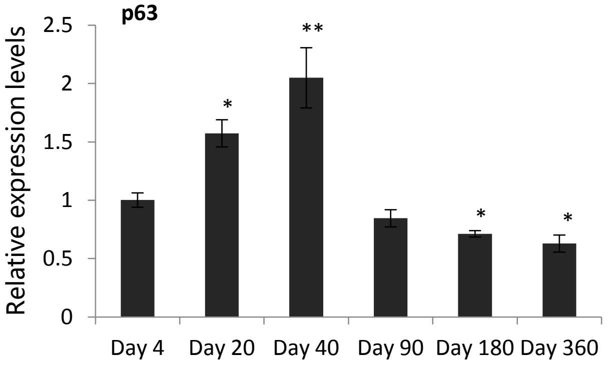

Supplement: Supplementary file 1 — Supplemental Data [file 41419_2022_4777_MOESM1_ESM.pdf]
